# Trends of childhood Composite Index of Anthropometric Failure prevalence, determinants and inequity in Bangladesh: insights from Bangladesh Demographic and Health Surveys

**DOI:** 10.1017/S1368980026101931

**Published:** 2026-02-04

**Authors:** Abu Sayeed, Nondo Saha, Aishi Aratrika, Ema Akter, Hassan Rushekh Mahmood, Lubna Hossain, Sahar Raza, Fariya Rahman, Shams El Arifeen, Ahmed Ehsanur Rahman, Anisuddin Ahmed, Syed Moshfiqur Rahman

**Affiliations:** 1 Maternal and Child Health Division (MCHD), International Centre for Diarrhoeal Disease Research Bangladesh (icddr,b), Dhaka, Bangladesh; 2 Department of Epidemiology, Biostatistics and Occupational Health, School of Population and Global Health, McGill University, Montreal, QC, Canada; 3 Department of Public Health, North South University, Dhaka, Bangladesh; 4 Global Health and Migration Unit, Department of Women’s and Children’s Health, Uppsala University, Uppsala, Sweden

**Keywords:** Bangladesh, BDHS, Children, Undernutrition, Stunting, Wasting, Underweight

## Abstract

**Objective:**

The childhood Composite Index of Anthropometric Failure (CIAF) effectively identifies multiple anthropometric deficits among under-five children. This study aimed to analyse undernutrition among under-five children, as measured by childhood CIAF, to evaluate trends, determinants and disparities in Bangladesh between 2007 and 2022.

**Design:**

The study utilised data from five rounds of the nationally representative cross-sectional Bangladesh Demographic and Health Survey (BDHS) conducted in 2007, 2011, 2014, 2017–2018 and 2022. The CIAF was estimated using six mutually exclusive anthropometric failure categories in accordance with the WHO child growth standards. Multivariable logistic regression was performed to explore determinants of CIAF. The concentration index and concentration curve were used to assess the changes in inequity.

**Setting:**

Bangladesh.

**Participants:**

32 096 under-five children.

**Results:**

The prevalence of childhood CIAF declined significantly from 56 % (95 % CI: 55, 58) in 2007 to 35 % (95 % CI: 33, 36) in 2022. The significant reduction in ‘stunting and underweight’ from 23 % (95 % CI: 22, 24) in 2007 to 11 % (95 % CI: 10, 12) in 2022 was a major contributor to the decrease in childhood CIAF. Child age, household socio-economic status (SES) and mother’s education were significant determinants of childhood CIAF across all study periods. Negative concentration indices for SES, residence and mother’s and father’s education indicate pro-poor inequality in childhood CIAF, which declined from 2007 to 2022.

**Conclusions:**

Despite significant progress, disparities in childhood CIAF across SES, residence and parental education persist in Bangladesh. Targeted policy interventions are crucial to mitigating childhood undernutrition and achieving Sustainable Development Goal 2.2.

Child malnutrition, encompassing both undernutrition and overnutrition, persistently remains as one of the most critical global health challenges despite significant improvements in targeted nutritional interventions. According to the Joint Child Malnutrition Estimates by UNICEF, the WHO and the World Bank, an estimated 148·1 million children under 5 years of age were stunted, 45 million children were wasted and 340 million children were overweight as of 2022^(1)^. The South Asian subregion carries a disproportionate burden of child malnutrition, with an approximately 8 percentage points higher stunting prevalence than the global average and almost twice the wasting rate (14·1 %) than the global average of 6·7 %^(2)^. Alarmingly, more than half of the children worldwide experiencing wasting and severe wasting reside in South Asia^(3)^.

In Bangladesh, child malnutrition remains a critical concern. The prevalence of stunting among children under 5 years of age was estimated to be 28·0 % in 2020, which was considerably higher than the regional average of 21·8 % in Asia. Although the prevalence of wasting slightly declined from 16·6 % in 2000 to 9·8 % in 2020, it remained above the regional average of 8·9 %^(4)^. Over the past decade, Bangladesh has experienced a significant reduction in the prevalence of underweight children, from 39·8 % to 22·5 %^(5)^. However, the country is considered off-track in preventing the rising prevalence of overweight in children^(4)^. Although Bangladesh has made considerable progress in reducing child malnutrition, continued efforts are required to address the remaining higher levels of stunting and wasting.

Children with multiple concurrent anthropometric deficits are at an elevated risk of morbidity and mortality^(6)^. However, the overall burden of these overlapping deficits may not be fully captured by individual anthropometric indicators such as stunting, wasting and underweight, as they are not mutually exclusive and can exist together^(7)^. The Composite Index of Anthropometric Failure (CIAF) combines three conventional anthropometric indices (underweight, stunting and wasting) for children under 5 years of age and is particularly effective at identifying children with multiple anthropometric failures (Table 1)^(8)^. It was first introduced by Peter Svedberg in 2000 to estimate the overall prevalence of child nutritional status by combining these three standard indicators into a single index comprising six subgroups, from ‘A’ indicating no failure to ‘F’ denoting stunting only^(9)^. Nandy et al. provided the first detailed CIAF estimates using data from the Indian National Family Health Survey (NFHS) in 2005, with the addition of a seventh subgroup, ‘Y’ for underweight only^(10)^. In recent years, CIAF has gained increasing traction as a nutritional assessment tool for evaluating child malnutrition in Bangladesh. A study by Islam et al. (2019), one of the early applications of CIAF in Bangladesh, highlighted substantial levels of undernutrition with an estimated prevalence of 48·3 % (95 % CI (47·1 %, 49·5 %)) among children under 5 years of age.

**Table 1.** Classification of Composite Index of Anthropometric Failure (CIAF) among under-five children

Group	Description	Wasting	Stunting	Underweight
A	No failure	X	X	X
B	Wasting only	√	X	X
C	Wasting and underweight	√	X	√
D	Wasting, stunting and underweight	√	√	√
E	Stunting and underweight	X	√	√
F	Stunting only	X	√	X
Y	Underweight only	X	X	√

NAF, no anthropometric failure; CIAF, Composite Index of Anthropometric Failure.

NAF = A; CIAF = sum of B, C, D, E, F, Y.

CIAF offers a singular, comprehensive estimate of multiple overlapping anthropometric deficits, thereby mitigating the underestimation inherent in individual indicators. Since CIAF provides a single estimate, it captures a nuanced, composite picture of multiple overlapping anthropometric deficits, reduces the underestimation of varying levels of malnutrition in the population and aligns with Sustainable Development Goal 2.2, which aims to eradicate all forms of malnutrition by 2030^(11,12)^. Therefore, in specific nutritional evaluation settings requiring a more detailed assessment, CIAF can be a valuable complementary tool alongside conventional and globally established child nutrition indicators by WHO.

Despite multiple studies using conventional single anthropometric indicators using the previous Bangladesh Demographic and Health Survey (BDHS), Bangladesh’s most recent childhood CIAF estimates from BDHS 2022 have not yet been reported. In addition, a comprehensive trend analysis of childhood CIAF in Bangladesh over the years and its relevant covariates has yet to be explored. Therefore, this study aims to estimate trends in childhood CIAF prevalence, determinants and disparities in Bangladesh using data from five consecutive BDHS surveys. The findings from this study are expected to provide crucial insights into the overall burden of childhood multiple anthropometric failure in Bangladesh and how it changed over time, which, in turn, will inform more targeted and effective nutrition-related interventions to attain SDG 2.2.

## Methods

### Study design and sample

The present study utilised data from five rounds of the nationally representative cross-sectional BDHS conducted in 2007, 2011, 2014, 2017–2018, and 2022. Data are publicly accessible to registered users through the DHS website (www.dhsprogram.com). The surveys utilise a stratified, multi-stage (cluster), random sampling approach. Data on socio-economic, demographic, environmental and health characteristics of households was obtained by interviewing women aged 15–49 years, along with collecting the anthropometric measurements for children under 5 years of age. These nationally representative surveys collect data on population, health, nutrition and socio-economic indicators, emphasising maternal and child health. A standard questionnaire was used for data collection. Further details on methodology and findings are available in the respective BDHS reports^(13–17)^.

**Figure 1.**
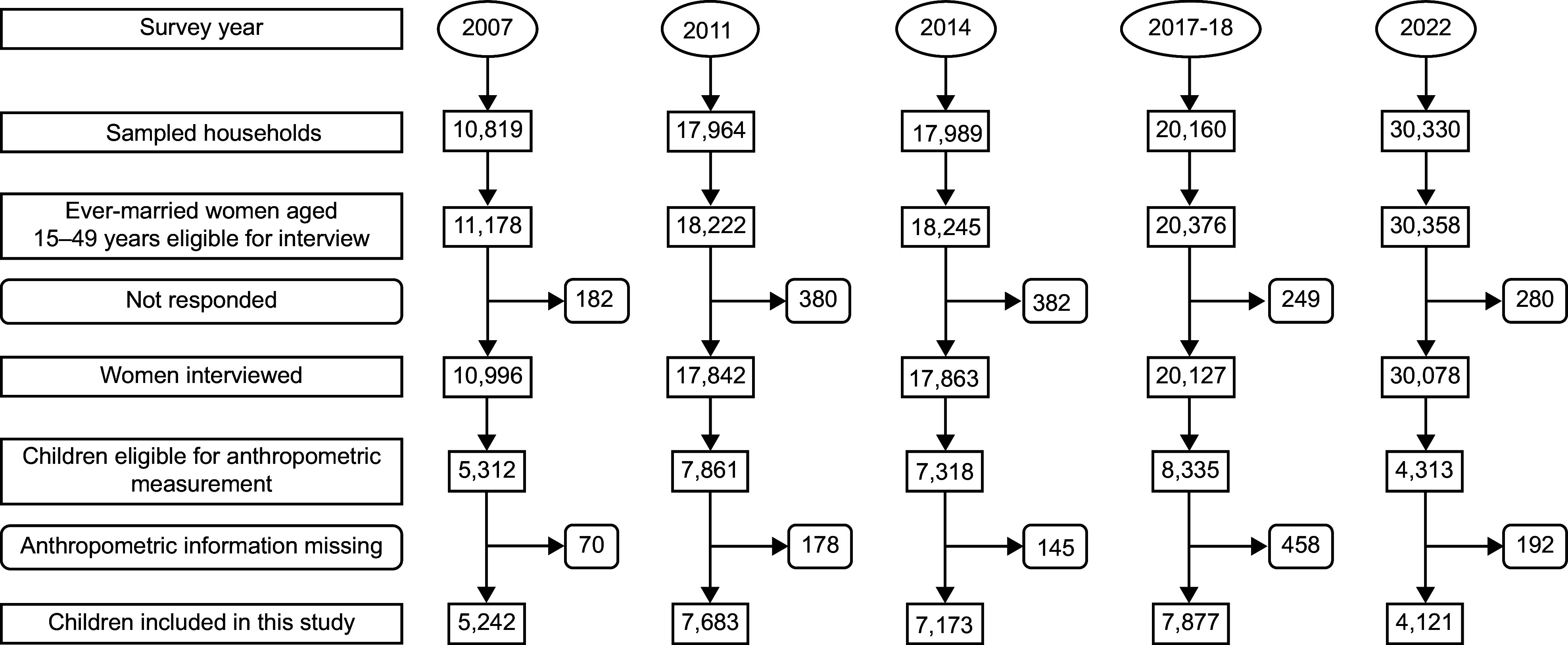
Study sample selection process.

This study was conducted based on a total of 32 096 children aged 0–59 months, comprising 5242 from 2007, 7683 from 2011, 7173 from 2014, 7877 from 2017–2018 and 4221 children from the 2022 BDHS, respectively (Figure 1). For the sampling frame classification, 2001 and 2011 population and housing censuses were used.

### Outcome measures and operational definitions

The primary outcome of the study was childhood undernutrition as represented by the CIAF, which was estimated using nutritional indicators of stunting, wasting and underweight. This assessment involved two steps. First, children were classified as stunted, wasted or underweight if their height-for-age, weight-for-height and weight-for-age fell below -2 sd, respectively, based on the WHO 2006 Growth Standards^(18)^. All the BDHS datasets analysed in this study provide children’s height-for-age, weight-for-height and weight-for-age information based on this standard. In the subsequent step, stunted, wasted and underweight children have been aggregated to determine the overall proportion of undernutrition. This aggregation results in seven distinct categories of A to Y: (A) no failure; (B) wasting only; (C) wasting and underweight; (D) wasting, stunting and underweight; (E) stunting and underweight; (F) stunting only; and (Y) underweight only (Table 1). Therefore, a child is considered as undernourished if he or she is suffering from any anthropometric failure (B–Y) above, coded as ‘1’, and no anthropometric failure (A) coded as ‘0’^(9)^.

### Explanatory variables

The independent variables for childhood undernutrition were selected based on previous literature^(19–21)^. These variables were classified into three categories according to UNICEF’s conceptual framework of malnutrition, including immediate (individual-level factors), underlying (household-level factors) and basic (community-level factors) determinants^(22)^. The individual-level determinants included child characteristics (child age, sex, birth order, birth interval and diarrhoea in the 2 weeks preceding the survey) and parental characteristics (maternal age, education, nutritional status and father’s education). The household-level covariates included socio-economic status (SES) as measured by wealth quintiles. The community-level factors include the place of residence, division and survey year^(13–17)^. Given the complexity in generating data on actual household income, BDHS constructed household SES from selected key household assets. The SES was computed by using principal component analysis, which assigns weights or factor scores to various indicators, and then standardises and sums for each household. The entire sample was subsequently ranked and divided into five equal groups, known as wealth quintiles, ranging from the first quintile (Q1, representing the poorest 20 %) to the fifth quintile (Q5, representing the richest 20 %)^(23)^.

### Data management and analysis

All statistical analyses were performed in Stata version 17.0 (StataCorp). Prior to analysis, influential outliers and missing observations were identified and excluded (Figure 1). The socio-demographic characteristics of the children were calculated by the use of descriptive statistics such as frequencies and proportions. Cross-tabulation was also applied for measuring the prevalence of CIAF concerning the independent variables. The association between independent and outcome variables was analysed using multivariate logistic regression analysis. Multi-collinearity diagnostic test was applied between the independent variables before logistic regression analysis. Decisive criteria were set out at a variance inflation factor value of < 5, and only variables within this limit were included in the multivariate logistic regression. Adjusted OR (AOR) with its corresponding 95 % CI was computed using multivariate logistic regression to identify the factors associated with childhood CIAF. The significance level in all analyses was set based on *P*-value and 95 % CI, with a threshold set at *P* < 0·05 (two-tailed). Sampling weights were utilised to adjust for unequal recruitment probabilities and ensure nationally representative estimates. To control the effect of the complex survey design, all the analyses of this study were performed using Stata’s *‘svy’* command.

### Concentration index and concentration curve

Econometric analysis was carried out using concentration index (CCI) and concentration curve (CC). CCI measured the inequality of outcome variables (CIAF) across SES, residence and parental education. The index ranges from − 1 to + 1, where the index value of 0 shows no inequality, and larger absolute values reflect greater inequality concentration^(24)^. The CC is obtained by plotting the cumulative proportion of CIAF on y-axis against the increasing percentage of the population ranked by the socio-economic indicator (e.g. SES) on x-axis. A curve above the line of equality (45-degree line) indicates negative CCI values, suggesting disproportionate concentration among the poor, and vice versa^(24)^. We used Stata commands *‘conindex’* and *‘clorenz’* to measure the CCI and to plot the CC, respectively.

## Result

### Estimated prevalence of childhood Composite Index of Anthropometric Failure

**Figure 2.**
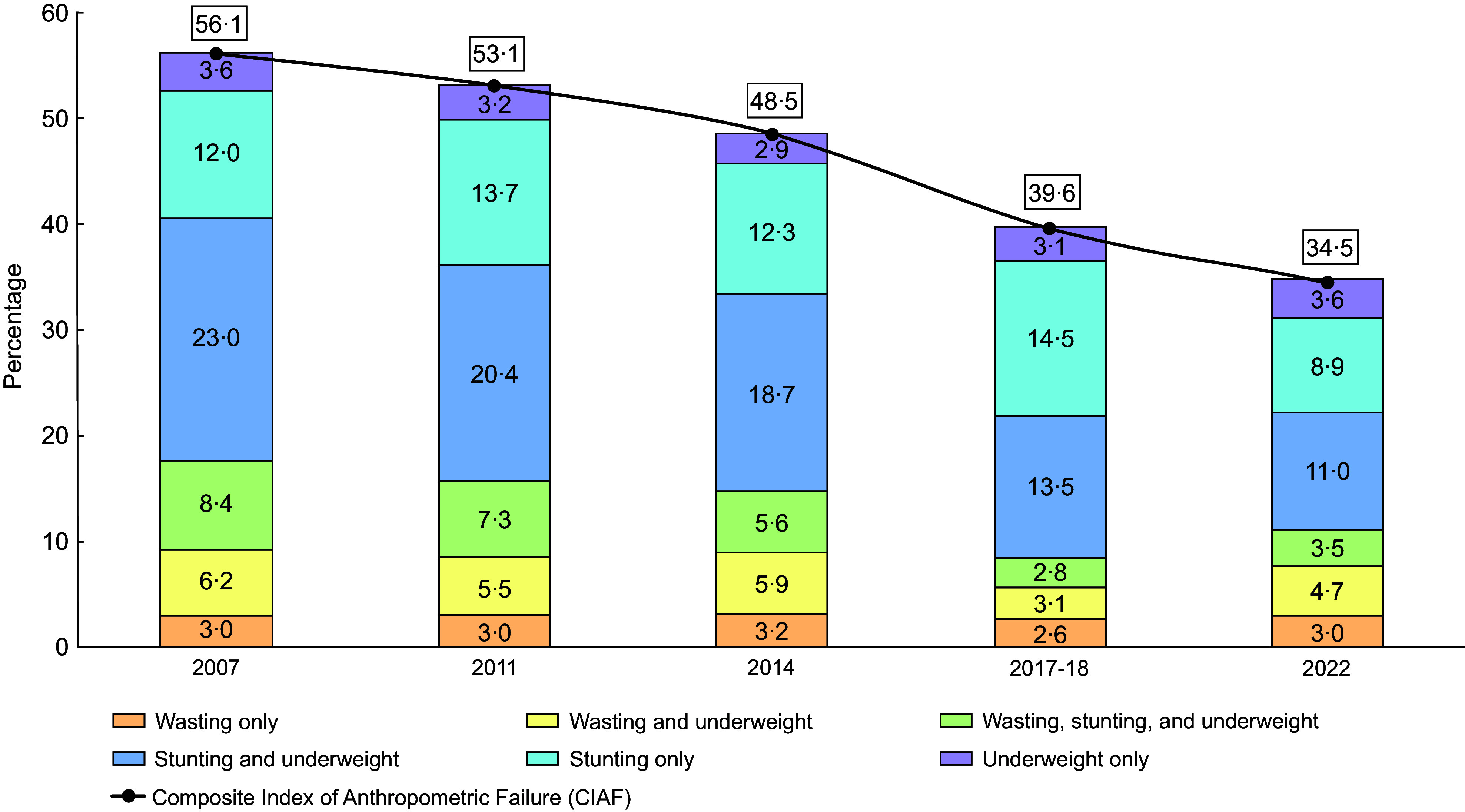
Trends in childhood CIAF in Bangladesh, 2007–2022. CIAF, Composite Index of Anthropometric Failure.

Figure 2 illustrates the trends in overall undernutrition status as measured by CIAF status among under-five children from 2007 to 2022. Childhood CIAF has shown a consistent decline over the periods. It decreased from 56·1 % in 2007 to 53·1 % in 2011, further dropping to 48·5 % in 2014, 39·6 % in 2017–2018 and finally reaching 34·5 % in 2022. The significant reduction in ‘stunting and underweight’ from 23 % (95 % CI: 22, 24) in 2007 to 11 % (95 % CI: 10, 12) in 2022 was a major contributor to the decrease in childhood CIAF. The prevalence of ‘only wasting’ (3·0 %) and ‘only underweight’ (3·6 %) remained unchanged between 2007 and 2022, while only stunting showed a slight decrease from 12 % in 2007 to 8·9 % in 2022.

Additionally, the distribution of under-five undernutrition based on the seven categories of CIAF classification has been summarised in online supplementary material, Supplemental Table 1. In 2007, 56·1 % of the children had one or more forms of CIAF, with stunting (43·4 %), wasting (17·6 %) and underweight (41·1 %). Over time, the prevalence of all anthropometric measures showed a decline, except for wasting and underweight in 2017–2018. By 2022, stunting was observed among 23·4 % of children, wasting in 11·2 % and underweight in 22·6 % (see online supplementary material, Supplemental Figure 1).

### Distribution of childhood Composite Index of Anthropometric Failure across socio-demographic and economic characteristics

A total of 32 096 under-five children were included to determine the prevalence, factors and inequity. Among the under-five children, the distribution of age remained relatively similar over the years. The proportion of children living in urban areas has gradually increased from 21·0 % in 2007 to 26·0 % in 2022. The detailed background characteristics of the participants are illustrated in online supplementary material, Supplemental Table 2.

Table 2 presents the estimated prevalence of childhood CIAF across socio-economic and demographic characteristics. The prevalence of CIAF declined over time across most factors, with consistent reductions in all age groups and both sexes. Among children aged less than 12 months, the prevalence of CIAF decreased substantially from 41·0 % in 2007 to 26·8 % in 2022. The CIAF remained higher among rural children, those in lower wealth quintiles, children of uneducated parents and those with higher birth orders. Rural areas consistently demonstrated higher CIAF rates than urban areas, though the rural–urban gap decreased from 10·2 % in 2007 to 1·8 % in 2022. Children born after short birth intervals (< 24 months) consistently had the highest rates of CIAF from 65·5 % in 2007 to 47·1 % in 2022. In terms of SES quintiles, CIAF prevalence among the poorest quintile declined from 67·4 % in 2007 to 46·2 % by 2022, while in the richest quintile this statistic decreased from 37·8 % to 27·5 %. Children from older mothers (35–49 years) had higher CIAF rates in 2007, 2011 and 2022. Children of mothers with no formal education exhibited the highest CIAF rates, decreasing from 64·0 % in 2007 to 55·3 % in 2022. Similarly, in 2022, the prevalence of CIAF among children of fathers with no formal education was 44·1 %, compared to 27·3 % among those whose fathers had attained higher education. Children born to malnourished mothers or those who experienced diarrhoea had higher CIAF prevalence, although the estimates for both conditions declined over time (Table 2).

**Table 2.** Prevalence of childhood CIAF by socio-demographic and economic characteristics in Bangladesh from 2007 to 2022

Variables	2007			2011			2014			2017–18			2022		
	*n*/N	%	95 % CI	*n*/N	%	95 % CI	*n*/N	%	95 % CI	*n*/N	%	95 % CI	*n*/N	%	95 % CI
Age of child (months)															
Less than 12	429/1046	41·0	38·1, 44·0	556/1499	37·1	34·7, 39·6	522/1407	37·1	34·6, 39·7	511/1708	29·9	27·8, 32·1	249/932	26·8	24·0, 29·7
12–23	595/1075	55·4	52·4, 58·3	849/1442	58·9	56·3, 61·4	760/1542	49·3	46·8, 51·8	670/1623	41·3	38·9, 43·7	296/822	36·0	32·8, 39·4
24–35	657/1058	62·1	59·1, 65·0	824/1419	58·1	55·5, 60·6	750/1423	52·7	50·1, 55·3	729/1551	47·0	44·5, 49·5	311/798	38·9	35·6, 42·4
36–47	641/1018	63·0	60·0, 65·9	982/1704	57·7	55·3, 60·0	749/1401	53·5	50·9, 56·1	631/1478	42·7	40·2, 45·3	285/778	36·7	33·3, 40·1
48–59	620/1045	59·3	56·3, 62·3	871/1619	53·8	51·4, 56·2	700/1400	50·0	47·4, 52·6	575/1516	37·9	35·5, 40·4	280/790	35·4	32·2, 38·8
Sex of child															
Male	1468/2600	56·5	54·5, 58·4	2016/3905	51·6	50·1, 53·2	1840/3720	49·5	47·9, 51·1	1638/4107	39·9	38·4, 41·4	728/2108	34·5	32·5, 36·6
Female	1475/2641	55·8	53·9, 57·7	2066/3778	54·7	53·1, 56·3	1641/3453	47·5	45·9, 49·2	1479/3769	39·2	37·7, 40·8	694/2013	34·5	32·4, 36·6
Residence															
Urban	531/1102	48·1	45·2, 51·1	798/1700	46·9	44·6, 49·3	745/1807	41·2	39, 43·5	725/2080	34·9	32·8, 36·9	355/1071	33·2	30·4, 36·1
Rural	2412/4139	58·3	56·8, 59·8	3284/5982	54·9	53·6, 56·2	2737/5366	51·0	49·7, 52·3	2391/5797	41·3	40·0, 42·5	1066/3050	35·0	33·3, 36·7
Division															
Barisal	207/338	61·2	55·8, 66·2	236/416	56·8	51·9, 61·5	223/412	54·1	49·3, 58·9	185/445	41·7	37·2, 46·4	109/284	38·2	32·8, 44·1
Chittagong	652/1145	56·9	54·1, 59·8	927/1745	53·2	50·8, 55·5	799/1516	52·7	50·1, 55·2	665/1628	40·8	38·5, 43·3	315/913	34·5	31·5, 37·7
Dhaka	919/1664	55·2	52·8, 57·6	1319/2405	54·8	52·8, 56·8	1113/2517	44·2	42·3, 46·2	976/2631	37·1	35·3, 39·0	449/1347	33·3	30·8, 35·9
Khulna	248/504	49·2	44·8, 53·6	325/725	44·9	41·3, 48·5	226/546	41·4	37·4, 45·6	246/740	33·3	30·0, 36·8	121/410	29·5	25·3, 34·2
Rajshahi	662/1151	57·5	54·7, 60·4	909/1801	50·5	48·2, 52·8	715/1483	48·2	45·7, 50·8	697/1778	39·2	37·0, 41·5	291/860	33·8	30·7, 37·0
Sylhet	255/440	57·9	53·2, 62·4	366/591	61·8	57·8, 65·7	406/698	58·1	54·4, 61·7	346/655	52·8	49·0, 56·6	138/307	44·8	39·3, 50·5
Wealth quintile															
Poorest	790/1172	67·4	64·7, 70·0	1182/1810	65·3	63·1, 67·5	1006/1629	61·8	59·4, 64·1	850/1717	49·5	47·1, 51·8	393/851	46·2	42·9, 49·6
Poorer	710/1131	62·8	59·9, 65·5	936/1569	59·7	57·2, 62·1	757/1349	56·1	53·4, 58·7	736/1617	45·5	43·1, 47·9	325/842	38·7	35·4, 42·0
Middle	572/1018	56·2	53·1, 59·2	794/1493	53·2	50·7, 55·7	693/1422	48·7	46·1, 51·3	560/1503	37·3	34·9, 39·8	274/857	32·0	29·0, 35·2
Richer	519/989	52·5	49·3, 55·6	683/1465	46·6	44·1, 49·2	612/1427	42·9	40·3, 45·5	589/1581	37·3	34·9, 39·7	215/793	27·1	24·1, 30·3
Richest	353/932	37·8	34·8, 41·0	486/1345	36·2	33·6, 38·8	413/1345	30·7	28·3, 33·3	381/1459	26·1	23·9, 28·5	214/778	27·5	24·5, 30·7
Birth order															
First	875/1725	50·7	48·4, 53·1	1330/2667	49·9	48·0, 51·8	1218/2754	44·2	42·4, 46·1	1111/3002	37·0	35·3, 38·8	490/1562	31·3	29·1, 33·7
Second	769/1388	55·4	52·8, 58·0	1133/2240	50·6	48·5, 52·7	1047/2155	48·6	46·5, 50·7	971/2560	37·9	36·1, 39·8	477/1418	33·6	31·2, 36·1
Third	515/901	57·2	54·0, 60·4	702/1337	52·5	49·8, 55·1	577/1174	49·1	46·3, 52·0	548/1330	41·2	38·6, 43·8	267/740	36·0	32·6, 39·5
Fourth or more	784/1229	63·8	61·1, 66·5	917/1439	63·8	61·3, 66·2	640/1089	58·7	55·8, 61·6	487/984	49·4	46·3, 52·6	189/401	47·1	42·2, 52·0
Birth interval															
First birth	884/1739	50·8	48·5, 53·2	1352/2699	50·1	48·2, 52·0	1235/2782	44·4	42·6, 46·3	1135/3041	37·3	35·6, 39·1	494/1581	31·3	29·0, 33·6
Less than 24	306/468	65·5	61·0, 69·7	346/567	61·1	57·0, 65·0	289/485	59·5	55·1, 63·8	223/491	45·5	41·1, 49·9	121/257	47·1	41·0, 53·2
24–47	914/1466	62·4	59·9, 64·8	1153/1945	59·3	57·1, 61·4	817/1464	55·9	53·3, 58·4	642/1450	44·3	41·7, 46·8	259/626	41·4	37·6, 45·3
48 or more	831/1559	53·3	50·8, 55·8	1222/2452	49·9	47·9, 51·8	1135/2428	46·8	44·8, 48·7	1107/2875	38·5	36·8, 40·3	541/1647	32·9	30·6, 35·2
Diarrhoeal status															
No	2606/4713	55·3	53·9, 56·7	3847/7322	52·6	51·4, 53·7	3258/6756	48·2	47·0, 49·4	2976/7493	39·7	38·6, 40·8	1359/3903	34·8	33·3, 36·3
Yes	336/528	63·6	59·4, 67·6	233/359	64·9	59·8, 69·7	219/411	53·2	48·4, 58·0	140/379	36·8	32·1, 41·8	62/212	29·4	23·7, 36·0
Mother’s age															
15–19	425/791	53·7	50·2, 57·2	553/1029	53·8	50·7, 56·8	511/1050	48·7	45·7, 51·7	413/1020	40·4	37·5, 43·5	121/405	30·0	25·7, 34·6
20–34	2182/3933	55·5	53·9, 57·0	3163/6005	52·7	51·4, 53·9	2695/5575	48·3	47·0, 49·7	2454/6257	39·2	38·0, 40·4	1117/3263	34·3	32·6, 35·9
35–49	336/518	64·9	60·6, 68·9	365/649	56·3	52·4, 60·1	275/547	50·2	46·0, 54·4	250/600	41·7	37·8, 45·7	183/454	40·4	35·9, 45·0
Maternal education															
No formal education	899/1405	64·0	61·5, 66·5	990/1539	64·3	61·9, 66·7	682/1169	58·4	55·5, 61·2	306/567	54·0	49·9, 58·1	142/257	55·3	49·2, 61·3
Primary	1028/1658	62·0	59·6, 64·3	1410/2356	59·9	57·9, 61·8	1143/2008	56·9	54·8, 59·1	1075/2264	47·5	45·5, 49·6	357/893	40·0	36·8, 43·3
Secondary	908/1840	49·3	47·1, 51·6	1520/3252	46·7	45·0, 48·5	1447/3324	43·5	41·9, 45·2	1462/3843	38·0	36·5, 39·6	740/2242	33·0	31·1, 35·0
Higher	107/338	31·8	27·0, 37·0	162/536	30·2	26·5, 34·3	208/671	31·0	27·7, 34·7	272/1202	22·7	20·4, 25·1	182/729	25·0	22·0, 28·3
Maternal employment status															
Unemployed	2095/3842	54·5	53·0, 56·1	3723/6983	53·3	52·2, 54·5	2451/5291	46·3	45·0, 47·7	1768/4683	37·8	36·4, 39·2	1054/3075	34·3	32·6, 36
Employed	848/1399	60·6	58·0, 63·1	359/700	51·2	47·5, 54·9	1031/1881	54·8	52·5, 57·0	1348/3194	42·2	40·5, 43·9	367/1047	35·1	32·3, 38·1
Maternal nutritional status															
Normal	1730/3160	54·8	53, 56·5	2355/4618	51·0	49·6, 52·4	2113/4265	49·6	48·1, 51·1	1916/4734	40·5	39·1, 41·9	839/2330	36·0	34·1, 38·0
Malnourished	1203/2068	58·2	56, 60·3	1705/3021	56·4	54·7, 58·2	1349/2882	46·8	45·0, 48·6	1186/3116	38·1	36·4, 39·8	582/1790	32·5	30·4, 34·7
Father’s education															
No formal education	1191/1838	64·8	62·6, 67·0	1428/2280	62·6	60·6, 64·6	1102/1850	59·6	57·3, 61·8	606/1157	52·4	49·5, 55·3	275/622	44·1	40·3, 48·1
Primary	859/1467	58·5	56·0, 61·0	1296/2233	58·0	56·0, 60·1	1131/2160	52·4	50·3, 54·5	1192/2689	44·3	42·5, 46·2	457/1240	36·9	34·2, 39·6
Secondary	686/1349	50·9	48·2, 53·5	1027/2199	46·7	44·6, 48·8	920/2191	42·0	39·9, 44·1	932/2564	36·3	34·5, 38·2	455/1438	31·6	29·3, 34·1
Higher	205/581	35·2	31·4, 39·2	331/965	34·3	31·3, 37·3	328/970	33·8	30·9, 36·9	327/1326	24·7	22·4, 27·1	208/762	27·3	24·3, 30·6
Overall	2943/5242	56·1	54·8, 57·5	4082/7683	53·1	52·0, 54·3	3481/7173	48·5	47·4, 49·7	3116/7877	39·6	38·5, 40·7	1422/4121	34·5	33·1, 36·0

CIAF, Composite Index of Anthropometric Failure.

### Factors associated with childhood Composite Index of Anthropometric Failure

Table 3 presents AOR from multivariate logistic regression models assessing factors associated with childhood CIAF from 2007 to 2022. Child’s age, household SES and maternal education were consistently significant predictors across all survey periods. Children of all age groups between 12 and 59 months exhibited significantly higher odds of CIAF compared to those under 12 months. Sex differences were also observed in different periods. In 2014, female children possessed 10 % (AOR: 0·90, 95 % CI: 0·82, 0·99) lower odds of CIAF compared to male children. However, in 2011, female children were 12 % (AOR: 1·12, 95 % CI: 1·02, 1·23) more likely to be undernourished than their male counterparts. Regarding household SES, an inverse association was observed between household SES and childhood CIAF. Compared to children from the poorest households, those from the richest quintile had significantly lower odds of CIAF. Compared to children living in households with the poorest SES, the odds of being CIAF among children living in households with the richest SES were decreased by 55 % in 2007, 57 % in 2011, 60 % in 2014, 43 % in 2017–2018 and 44 % in 2022. Birth interval was also significantly associated with childhood CIAF in 2011, 2017–2018 and 2022. In addition, higher levels of maternal education were consistently associated with lower CIAF prevalence, with the greatest reduction observed among mothers with secondary or higher education. The odds of being CIAF among under-five children whose mothers attended higher education were 34 % (AOR: 0·66, 95 % CI: 0·47, 0·92), 43 % (AOR: 0·57, 95 % CI: 0·43, 0·75), 31 % (AOR: 0·69, 95 % CI: 0·53, 0·90), 55 % (AOR: 0·45, 95 % CI: 0·34, 0·58) and 57 % (AOR: 0·43, 95 % CI: 0·30, 0·63), less compared to children whose mother had no formal education in 2007, 2011, 2014, 2017–2018 and 2022, respectively. Mother’s nutritional status and father’s education were also significantly associated with childhood CIAF during multiple survey periods (Table 3).

**Table 3.** Estimated adjusted OR (AOR) for multivariate logistic regression models of childhood CIAF

Variables	2007			2011			2014			2017–18			2022		
	AOR	95 % CI	*P*-value	AOR	95 % CI	*P*-value	AOR	95 % CI	*P*-value	AOR	95 % CI	*P*-value	AOR	95 % CI	*P*-value
Age of child in months (ref: less than 12)															
12–23	1·80	1·50, 2·15	**< 0·001**	2·63	2·25, 3·07	**< 0·001**	1·72	1·48, 2·01	**< 0·001**	1·70	1·47, 1·98	**< 0·001**	1·54	1·25, 1·91	**< 0·001**
24–35	2·37	1·97, 2·85	**< 0·001**	2·55	2·18, 2·99	**< 0·001**	2·00	1·71, 2·35	**< 0·001**	2·12	1·82, 2·47	**< 0·001**	1·68	1·36, 2·08	**< 0·001**
36–47	2·34	1·94, 2·82	**< 0·001**	2·51	2·15, 2·92	**< 0·001**	2·04	1·73, 2·40	**< 0·001**	1·74	1·48, 2·03	**< 0·001**	1·55	1·24, 1·93	**< 0·001**
48–59	1·98	1·64, 2·39	**< 0·001**	2·11	1·80, 2·46	**< 0·001**	1·67	1·42, 1·97	**< 0·001**	1·40	1·19, 1·64	**< 0·001**	1·38	1·11, 1·73	**0·004**
Sex of child (ref: male)															
Female	0·95	0·85, 1·07	0·426	1·12	1·02, 1·23	**0·022**	0·90	0·82, 0·99	**0·032**	0·98	0·89, 1·07	0·630	1·01	0·88, 1·15	0·918
Residence (ref: urban)															
Rural	1·02	0·86, 1·20	0·818	0·87	0·76, 1·00	**0·047**	0·93	0·82, 1·06	0·303	0·96	0·85, 1·09	0·555	0·82	0·69, 0·97	**0·019**
Division (ref: Dhaka)															
Barisal	1·15	0·89, 1·48	0·280	0·99	0·79, 1·24	0·941	1·42	1·13, 1·77	**0·002**	1·14	0·92, 1·42	0·241	1·16	0·88, 1·54	0·292
Chittagong	1·13	0·96, 1·33	0·139	0·94	0·82, 1·07	0·333	1·46	1·28, 1·68	**< 0·001**	1·15	1·01, 1·32	**0·037**	0·96	0·79, 1·16	0·656
Khulna	0·84	0·68, 1·33	0·121	0·73	0·61, 0·87	**0·001**	0·89	0·73, 1·09	0·257	0·86	0·72, 1·04	0·113	0·91	0·71, 1·18	0·481
Rajshahi	1·10	0·93, 1·29	0·276	0·75	0·65, 0·85	**< 0·001**	1·02	0·89, 1·18	0·732	1·00	0·87, 1·14	0·966	1·00	0·83, 1·22	0·966
Sylhet	0·95	0·75, 1·19	0·633	1·16	0·95, 1·42	0·134	1·40	1·17, 1·68	**< 0·00**1	1·57	1·31, 1·89	**< 0·001**	1·32	1·01, 1·74	**0·042**
Wealth quintile (ref: poorest)															
Poorer	0·90	0·75, 1·07	0·240	0·89	0·77, 1·03	0·117	0·89	0·77, 1·04	0·145	0·95	0·82, 1·09	0·453	0·86	0·70, 1·05	0·145
Middle	0·71	0·58, 0·85	**< 0·001**	0·75	0·64, 0·87	**< 0·001**	0·72	0·62, 0·85	**< 0·001**	0·75	0·65, 0·88	**< 0·001**	0·64	0·52, 0·79	**< 0·001**
Richer	0·69	0·57, 0·85	**< 0·001**	0·61	0·52, 0·72	**< 0·001**	0·58	0·49, 0·69	**< 0·001**	0·80	0·68, 0·94	**0·007**	0·54	0·42, 0·68	**< 0·001**
Richest	0·45	0·35, 0·57	**< 0·001**	0·43	0·35, 0·52	**< 0·001**	0·40	0·33, 0·49	**< 0·001**	0·57	0·47, 0·70	**< 0·001**	0·56	0·43, 0·73	**< 0·001**
Birth order (ref: first)															
Second	2·09	0·68, 6·46	0·198	1·93	0·87, 4·30	0·108	1·98	0·85, 4·59	0·113	2·00	1·01, 3·95	**0·046**	0·52	0·17, 1·59	0·249
Third	1·99	0·64, 6·18	0·235	1·87	0·84, 4·16	0·128	1·75	0·75, 4·08	0·197	2·00	1·01, 3·98	**0·048**	0·53	0·17, 1·64	0·267
Fourth or more	2·02	0·65, 6·26	0·224	2·45	1·09, 5·51	**0·030**	2·11	0·90, 4·97	**0·087**	2·24	1·12, 4·48	**0·023**	0·68	0·22, 2·14	0·514
Birth interval (ref: first birth)															
Less than 24	0·70	0·22, 2·16	0·531	0·61	0·27, 1·37	0·230	0·79	0·33, 1·85	0·583	0·56	0·28, 1·12	0·102	2·91	0·92, 9·14	0·068
24–47	0·61	0·20, 1·87	0·386	0·57	0·26, 1·27	0·170	0·67	0·29, 1·55	0·345	0·52	0·26, 1·03	0·062	2·39	0·77, 7·40	0·13
48 or more	0·44	0·14, 1·37	0·157	0·46	0·21, 1·03	0·059	0·53	0·23, 1·22	0·133	0·47	0·24, 0·92	**0·027**	1·78	0·58, 5·48	0·312
Diarrhoeal status (ref: no)															
Yes	1·39	1·14, 1·69	**0·001**	1·61	1·27, 2·04	**< 0·001**	1·24	1·00, 1·53	**0·048**	0·87	0·70, 1·09	0·239	0·79	0·58, 1·09	0·149
Mother’s age (ref: 15–19)															
20–34	0·96	0·80, 1·16	0·705	0·81	0·69, 0·96	**0·013**	0·79	0·67, 0·93	**0·004**	0·90	0·76, 1·06	0·214	1·09	0·84, 1·42	0·510
35–49	1·25	0·93, 1·69	0·145	0·69	0·53, 0·90	**0·006**	0·66	0·50, 0·86	**0·002**	0·82	0·63, 1·08	0·156	1·13	0·79, 1·62	0·503
Maternal education (ref: no formal education)															
Primary	1·10	0·94, 1·30	0·234	0·96	0·83, 1·12	0·627	1·05	0·90, 1·24	0·514	0·82	0·67, 1·00	**0·049**	0·61	0·45, 0·82	**0·001**
Secondary	0·87	0·72, 1·05	0·141	0·81	0·69, 0·95	**0·011**	0·81	0·69, 0·96	**0·017**	0·69	0·56, 0·85	**0·001**	0·57	0·42, 0·76	**< 0·001**
Higher	0·66	0·47, 0·92	**0·015**	0·57	0·43, 0·75	**< 0·001**	0·69	0·53, 0·90	**0·006**	0·45	0·34, 0·58	**< 0·001**	0·43	0·30, 0·63	**< 0·001**
Maternal employment status (ref: unemployed)															
Employed	1·07	0·93, 1·22	0·339	0·93	0·78, 1·10	0·374	1·28	1·14, 1·43	**< 0·001**	1·04	0·94, 1·15	0·456	0·94	0·80, 1·10	0·461
Maternal nutritional status (ref: normal)															
Malnourished	1·12	1·00, 1·26	0·055	1·19	1·08, 1·31	**0·001**	0·94	0·85, 1·04	0·254	0·99	0·90, 1·09	0·831	0·85	0·74, 0·98	**0·021**
Father’s education (ref: no formal education)															
Primary	0·87	0·74, 1·01	0·064	1·00	0·87, 1·14	0·959	0·86	0·75, 0·98	**0·027**	0·82	0·71, 0·96	**0·011**	0·86	0·70, 1·06	0·163
Secondary	0·84	0·71, 1·00	0·051	0·82	0·71, 0·95	**0·009**	0·76	0·65, 0·89	**0·001**	0·73	0·62, 0·87	**< 0·001**	0·86	0·69, 1·07	0·182
Higher	0·59	0·46, 0·77	**< 0·001**	0·66	0·54, 0·82	**< 0·001**	0·78	0·63, 0·96	**0·022**	0·60	0·48, 0·74	**< 0·001**	0·91	0·68, 1·22	0·527

CIAF, Composite Index of Anthropometric Failure.

Bold *P*-values denote statistically significant values.

### Estimated inequalities in childhood Composite Index of Anthropometric Failure

**Figure 3.**
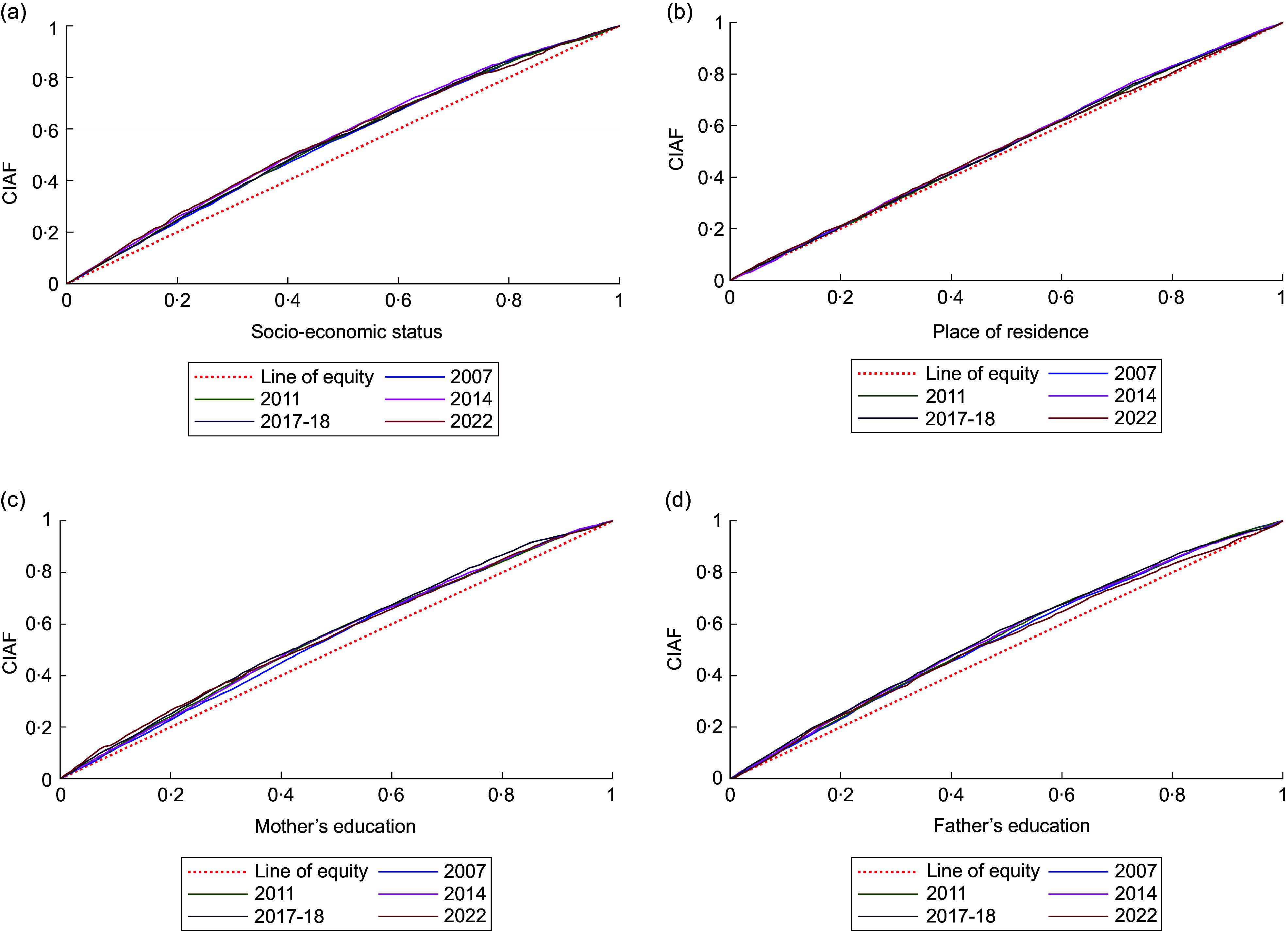
Disparities in childhood CIAF disaggregated by (a) socio-economic status, (b) place of residence, (c) mother’s education and (d) father’s education in Bangladesh (2007 to 2022). CIAF, Composite Index of Anthropometric Failure.

Figure 3 and online supplementary material, Supplemental Table 3A-3D, illustrate persistent inequalities in childhood undernutrition as measured by CIAF by household SES, residence and parental education. CC for all survey years lay above the equality line, indicating a disproportionate burden of CIAF among disadvantaged groups. This pattern is confirmed by consistently negative CCI values, reflecting greater CIAF prevalence among children from lower SES households, rural residence and parents with lower levels of education. Socio-economic inequality remained notable, with CCI values ranging from –0·220 (2007) to –0·175 (2022), peaking in 2014 (–0·241), though modest improvements followed. Residence-based disparities also declined, with CCI improving from –0·068 (2007) to –0·015 (2022), despite a peak in 2014 (–0·074). For maternal education, CIAF remained concentrated among less-educated mothers (CCI: –0.184 in 2007 to –0.153 in 2022), highest in 2011 (–0.204). Similar trends were observed for paternal education, with all CCI values negative, indicating consistent inequality. Overall, while disparities persist, recent data suggest a gradual reduction in undernutrition-related inequities over time. The pro-poor inequality in CIAF was statistically significant for all four factors – SES, residence, mother’s education and father’s education – across all years, with the exception of residence in 2022, where the difference between urban and rural children was no longer significant.

## Discussion

This study presents novel estimates on the prevalence, contributing factors and disparities related to childhood undernutrition by aggregating conventional undernutrition indicators and applying them to the BDHS dataset. Our findings suggest that individual measures of stunting, wasting and underweight underestimate the overall burden of undernutrition. Furthermore, prevalence estimates cannot be added to fully capture the extent of undernutrition, as children often present with multiple anthropometric deficits. The use of CIAF allows for the identification of all undernourished children, providing a comprehensive estimate of the undernutrition burden within a population^(11)^. The present study employed the CIAF for the first time to provide a comprehensive assessment of undernutrition among children under 5 years of age in Bangladesh over a 15-year period (2007–2022). The results show that the prevalence of undernutrition, presented as childhood CIAF, has declined steadily throughout the study periods. Factors such as child’s age, household SES and mother’s education were significantly associated with childhood CIAF across all study periods, while various other factors contributed to CIAF during specific time frames. CIAF was found to be more prevalent among children from the poorest wealth quintile, those living in rural areas and those with lower parental education levels; however, these inequities considerably declined over time.

Even though the prevalence of childhood undernutrition based on CIAF in Bangladesh has significantly decreased from 56 % to 35 % over time, it remains higher than in countries such as China (22 %)^(25)^ and Yemen (21 %)^(7)^, and it is lower than in studies conducted in India (48 %)^(26)^, Ethiopia (49 %)^(27)^, Tanzania (38 %)^(28)^ and Malawi (57 %)^(29)^. Two studies conducted in Bangladesh reported that the overall prevalence of undernutrition among children under 5 years of age, as assessed by childhood CIAF, was 48 % in 2014^(30)^ and 52 % in 2021^(31)^, respectively. The Government of Bangladesh targets to decrease the stunting burden to 25 % by the end of 2025. Implementing a comprehensive community-based intervention programme is crucial for effectively addressing undernutrition^(32)^. Our research findings align with previous studies indicating that undernutrition is more prevalent among rural children under 5 years of age compared to their urban counterparts. However, similar urban–rural disparities in undernutrition have not been consistently documented in other developing countries, except for India and Bangladesh^(33,34)^. A community-based study in Bangladesh found that undernutrition affects 48 % of children in rural areas and 58 % in urban areas^(34)^ in 2014. Similarly, in India, the prevalence of undernutrition, as measured by CIAF, was 54 % in urban regions and 64 % in rural regions^(32)^. Urban areas in Bangladesh have seen significant progress in recent years, including universal primary education, improved SES, better maternal healthcare, enhanced transportation and increased nutrition awareness, contributing to improved urban nutritional status^(35)^. In contrast, rural areas have also experienced substantial progress, with more than a two-thirds reduction of CIAF, reflecting nationwide improvements in health and nutrition programmes. Despite these gains, rural children continue to exhibit slightly higher CIAF rates compared with their urban counterparts in earlier survey years, likely due to slower development in infrastructure, access to healthcare and nutrition-related awareness^(36)^.

The present study explored that children under 12 months demonstrated lower odds of childhood CIAF compared to older children. This finding aligns with studies conducted in different countries like Tanzania^(28)^ and Yemen^(7)^. This trend may be attributed to younger children often receiving a more nutritious and balanced diet during early life stages, but as a child grows older, the discontinuation of breast-feeding and increased nutritional demands could contribute to higher vulnerability to malnutrition^(37)^. Additionally, younger children often receive greater attention and feeding effort from parents compared to their older siblings^(37)^. Furthermore, this study reveals that children from lower-income households are more likely to be affected by the CIAF compared to their higher-income counterparts. These findings are consistent with previous studies conducted in Ethiopia^(27)^ and Malawi^(29)^. Our findings also align with previous studies conducted in other developing countries^(32,38)^. This might be because the wealthiest households afford to purchase diverse and sufficient food supplies and have greater access to healthcare services, whereas poorer households face limitations in securing these necessities, contributing to undernutrition among children. Additionally, the analysis revealed that male children consistently had higher odds of being malnourished compared to their female counterparts in multiple survey periods. This underscores the role of gender in childhood undernutrition in Bangladesh and highlights the need for sex-specific nutritional interventions to promote optimal child growth and development. This finding is also consistent with a recent global systematic review and meta-analysis of forty-four studies, which reported a higher risk of stunting, wasting and being underweight among boys compared to girls^(39)^. Additionally, this study also aligns with pooled evidence from studies conducted between 2008 and 2020 across thirty-five sub-Saharan African countries, which reported that male children had higher odds of malnutrition^(40)^. This is also consistent with previous findings in India^(41)^ and Ghana^(42)^. Similarly, prior studies in Ethiopia^(43)^ and different low-income settings such as Kenya^(44)^, Tanzania^(28)^ and Indonesia^(45)^ have reported that being underweight is the most common undernutrition problem among boys compared to girls. This disparity is often attributed to biological growth patterns and the higher morbidity vulnerability of males in early infancy^(46)^. Besides, there is a perception that girls are less affected by environmental stress than boys^(47)^. According to our findings, caesarean delivery was associated with a lower likelihood of CIAF, consistent with findings from an Indian study^(26)^. Furthermore, we also found that childhood CIAF is significantly associated with maternal and paternal education. Children of lower-educated parents faced higher odds of CIAF than those with higher-educated parents. Consistent with studies in Tanzania^(28)^ and India^(26)^, maternal education was positively associated with improved child nutritional status. A Bangladeshi study conducted in 2020 also demonstrated that children of mothers with secondary or higher education experienced less growth failure^(32)^. Educated mothers are better equipped to adopt essential nutrition and hygiene practices and utilise information from educational resources and media, contributing to reduced anthropometric failures^(48)^. Similarly, fathers with formal education likely possess greater knowledge of proper child feeding and hygiene practices, which positively influence child nutrition outcomes. This finding aligns with previous studies conducted in sub-Saharan Africa^(28,49)^. Fathers with formal education might be more knowledgeable on appropriate child feeding and hygiene practices, contributing positively to the prevention of malnutrition and associated failures.

The present study also highlighted significant socio-economic-, residence- and education-related inequalities in childhood CIAF from 2007 to 2022. The CC consistently lying above the equity line indicate that CIAF is disproportionately concentrated among disadvantaged groups, with children from the poorest households, rural residence and lower-educated parents. The persistent negative CCIs underscore enduring socio-economic inequalities, with children from the poorest households continuing to bear a disproportionate burden of CIAF. Although 2014 marked the peak of socio-economic- and residence-related inequality, and 2011 marked the peak of education-related inequity, subsequent years show slight improvement, suggesting progress in reducing disparities. However, substantial inequalities remain. Contrary to our findings, a community-based study in Bangladesh indicated that socio-economic disparities in stunting have increased over time from 2007 to 2014^(31)^. Meanwhile, a global systematic review found that the relative gap in CIAF prevalence between the poorest and richest quintiles has decreased in low- and middle-income countries, including Bangladesh^(50)^. This could be attributed to the Government of Bangladesh’s targeted poverty reduction initiatives, such as the Social Safety Net Programmes (SSNP), as well as international collaborations with the World Food Programme and the FAO, which have improved access to nutrition, healthcare and education for vulnerable populations. These sustained efforts may have contributed to narrowing disparities and reducing inequality in recent years^(5)^. Additionally, rural–urban disparities in child CIAF persist, with rural children facing higher rates. However, the disparities narrowed over the periods, reflecting reduced inequity and the impact of targeted interventions. Furthermore, the consistently negative CI values for maternal and paternal education suggest that children of less-educated parents are more likely to experience CIAF, emphasising the need for policies that promote parental education, particularly among marginalised populations. To address these inequalities, socio-economic support programmes, rural-focused health initiatives and education campaigns for parents are required. These measures are essential to reduce disparities and improve child nutrition outcomes in Bangladesh.

### Strengths and limitations

To our knowledge, this study is the first of its kind that systematically investigates socio-economic inequality in childhood undernutrition prevalence using CIAF across multiple time periods for which comparable data are available. Additionally, we utilised the WHO child growth standard to measure the nutritional indicators stunting, wasting and underweight which are the basis for calculating CIAF. The further strength is that the data were collected from across the country, ensuring a nationally representative sample. In addition, using a nationwide population-based dataset provides a large sample size, enhancing the reliability and generalisability of the findings. Additionally, five consecutive surveys data provided a comparison of the findings over time. Furthermore, the application of UNICEF’s conceptual framework on the underlying causes of malnutrition facilitated a more precise assessment of the factors contributing to its burden. Several limitations should also be considered: first, since this study was based on cross-sectional data, it cannot establish a causal relationship between outcome and independent variables. Another limitation involves that the BDHS data were collected retrospectively and based on self-reporting, which may introduce underreporting, information bias and recall bias.

### Conclusion

This study highlights a comprehensive overview of trends in under-five undernutrition as measured by childhood CIAF in Bangladesh from 2007 to 2022. The findings reveal a significant decline in childhood CIAF in Bangladesh during this period, with a significant reduction in ‘stunting and underweight’. CIAF was significantly associated with child age, sex, SES and parental education. Although socio-economic-, residence- and education-related inequalities in CIAF persisted, their magnitude decreased over time. To further reduce inequities in undernutrition, it is essential to design and implement evidence-based, targeted and multisectoral strategies. In line with SDG 2.2, policymakers should prioritise integrated nutrition and health interventions to lower CIAF prevalence.

## Supporting information

Sayeed et al. supplementary material Sayeed et al. supplementary material

## Data Availability

The data underlying the results presented in the study are publicly accessible and available from the DHS website (http://dhsprogram.com/data/availabledatasets.cfm). The database has been accessed under a formal request and used exclusively for research purposes. The DHS had no role in the study design and data analysis. Additionally, de-identified data of the present analysis can be available from the corresponding author on reasonable request.

## Supplementary material

For supplementary material accompanying this paper visit https://doi.org/10.1017/S1368980026101931
