# Supplementary material for: Trends of childhood Composite Index of Anthropometric Failure prevalence, determinants and inequity in Bangladesh: insights from Bangladesh Demographic and Health Surveys
Source: Public Health Nutr. 2026 Feb 4;29(1):e67. doi: 10.1017/S1368980026101931 (PMC13087991; doi:10.1017/S1368980026101931)
Supplement: Sayeed et al. supplementary material [file S1368980026101931sup001.docx]

**Supplementary Table 1** Distribution of composite index of anthropometric failure (CIAF) of under-five children in Bangladesh from 2007 to 2022

| **Nutritional condition** | **CIAF Groups** | **2007** | | **2011** | | **2014** | | **2017-18** | | **2022** | |
| --- | --- | --- | --- | --- | --- | --- | --- | --- | --- | --- | --- |
|  |  | **n (%)** | **95% CI** | **n (%)** | **95% CI** | **n (%)** | **95% CI** | **n (%)** | **95% CI** | **n (%)** | **95% CI** |
| No anthropometric failure | A | 2299 (43.9) | (42.5, 45.2) | 3601 (46.9) | (45.8, 48.0) | 3691 (51.5) | (50.3, 52.6) | 4760 (60.4) | (59.4, 61.5) | 2699 (65.5) | (64.0, 66.9) |
| Composite index of anthropometric failure  (Sum of B, C, D, E, F, Y) |  | 2943 (56.1) | (54.8, 57.5) | 4082 (53.1) | (52.0, 54.3) | 3481 (48.5) | (47.4, 49.7) | 3116 (39.6) | (38.5, 40.7) | 1422 (34.5) | (33.1, 36.0) |
| Wasting only | B | 157 (3.0) | (2.6, 3.5) | 232  (3.0) | (2.7, 3.4) | 227 (3.2) | (2.8, 3.6) | 204 (2.6) | (2.3, 3.0) | 123 (3.0) | (2.5, 3.6) |
| Wasting and Underweight | C | 327 (6.2) | (5.6, 6.9) | 426  (5.5) | (5.1, 6.1) | 426 (5.9) | (5.4, 6.5) | 247 (3.1) | (2.8, 3.5) | 192 (4.7) | (4.1, 5.3) |
| Wasting, Stunting, and Underweight | D | 439 (8.4) | (7.7, 9.2) | 557  (7.3) | (6.7, 7.9) | 399 (5.6) | (5.1, 6.1) | 222 (2.8) | (2.5, 3.2) | 143 (3.5) | (2.9, 4.1) |
| Stunting and Underweight | E | 1205 (23.0) | (21.9, 24.1) | 1568 (20.4) | (19.5, 21.3) | 1339 (18.7) | (17.8, 19.6) | 1063 (13.5) | (12.8, 14.3) | 451 (11.0) | (10.0, 11.9) |
| Stunting only | F | 630 (12.0) | (11.2, 12.9) | 1053 (13.7) | (13.0, 14.5) | 884 (12.3) | (11.6, 13.1) | 1140 (14.5) | (13.7, 15.3) | 366 (8.9) | (8.1, 9.8) |
| Underweight only | Y | 186 (3.6) | (3.1, 4.1) | 246  (3.2) | (2.8, 3.6) | 207 (2.9) | (2.5, 3.3) | 241 (3.1) | (2.7, 3.5) | 147 (3.6) | (3.0, 4.2) |

**Supplementary Table 2** Socio-demographic and economic characteristics of study participants in Bangladesh, 2007-2022 (N=32,096)

| **Variables** | **2007** | | **2011** | | **2014** | | **2017-18** | | **2022** | |
| --- | --- | --- | --- | --- | --- | --- | --- | --- | --- | --- |
|  | **n** | **%** | **n** | **%** | **n** | **%** | **n** | **%** | **n** | **%** |
| **Age of child (months)** |  |  |  |  |  |  |  |  |  |  |
| Less than 12 | 1046 | 20.0 | 1499 | 19.5 | 1407 | 19.6 | 1708 | 21.7 | 932 | 22.6 |
| 12 to 23 | 1075 | 20.5 | 1442 | 18.8 | 1542 | 21.5 | 1623 | 20.6 | 822 | 20.0 |
| 24 to 35 | 1058 | 20.2 | 1419 | 18.5 | 1423 | 19.8 | 1551 | 19.7 | 798 | 19.4 |
| 36 to 47 | 1018 | 19.4 | 1704 | 22.2 | 1401 | 19.5 | 1478 | 18.8 | 778 | 18.9 |
| 48 to 59 | 1045 | 19.9 | 1619 | 21.1 | 1400 | 19.5 | 1516 | 19.3 | 790 | 19.2 |
| **Sex of child** |  |  |  |  |  |  |  |  |  |  |
| Male | 2600 | 49.6 | 3905 | 50.8 | 3720 | 51.9 | 4107 | 52.2 | 2108 | 51.1 |
| Female | 2641 | 50.4 | 3778 | 49.2 | 3453 | 48.1 | 3769 | 47.9 | 2013 | 48.9 |
| **Residence** |  |  |  |  |  |  |  |  |  |  |
| Urban | 1102 | 21.0 | 1700 | 22.1 | 1807 | 25.2 | 2080 | 26.4 | 1071 | 26.0 |
| Rural | 4139 | 79.0 | 5982 | 77.9 | 5366 | 74.8 | 5797 | 73.6 | 3050 | 74.0 |
| **Division** |  |  |  |  |  |  |  |  |  |  |
| Barisal | 338 | 6.4 | 416 | 5.4 | 412 | 5.7 | 445 | 5.6 | 284 | 6.9 |
| Chittagong | 1145 | 21.8 | 1745 | 22.7 | 1516 | 21.1 | 1628 | 20.7 | 913 | 22.2 |
| Dhaka | 1664 | 31.8 | 2405 | 31.3 | 2517 | 35.1 | 2631 | 33.4 | 1347 | 32.7 |
| Khulna | 504 | 9.6 | 725 | 9.4 | 546 | 7.6 | 740 | 9.4 | 410 | 10.0 |
| Rajshahi | 1151 | 22.0 | 1801 | 23.4 | 1483 | 20.7 | 1778 | 22.6 | 860 | 20.9 |
| Sylhet | 440 | 8.4 | 591 | 7.7 | 698 | 9.7 | 655 | 8.3 | 307 | 7.4 |
| **Wealth quintile** |  |  |  |  |  |  |  |  |  |  |
| Poorest | 1172 | 22.4 | 1810 | 23.6 | 1629 | 22.7 | 1717 | 21.8 | 851 | 20.7 |
| Poorer | 1131 | 21.6 | 1569 | 20.4 | 1349 | 18.8 | 1617 | 20.5 | 842 | 20.4 |
| Middle | 1018 | 19.4 | 1493 | 19.4 | 1422 | 19.8 | 1503 | 19.1 | 857 | 20.8 |
| Richer | 989 | 18.9 | 1465 | 19.1 | 1427 | 19.9 | 1581 | 20.1 | 793 | 19.2 |
| Richest | 932 | 17.8 | 1345 | 17.5 | 1345 | 18.8 | 1459 | 18.5 | 778 | 18.9 |
| **Birth order** |  |  |  |  |  |  |  |  |  |  |
| First | 1725 | 32.9 | 2667 | 34.7 | 2754 | 38.4 | 3002 | 38.1 | 1562 | 37.9 |
| Second | 1388 | 26.5 | 2240 | 29.2 | 2155 | 30.0 | 2560 | 32.5 | 1418 | 34.4 |
| Third | 901 | 17.2 | 1337 | 17.4 | 1174 | 16.4 | 1330 | 16.9 | 740 | 18.0 |
| Fourth or more | 1229 | 23.4 | 1439 | 18.7 | 1089 | 15.2 | 984 | 12.5 | 401 | 9.7 |
| **Birth interval** |  |  |  |  |  |  |  |  |  |  |
| First birth | 1739 | 33.3 | 2699 | 35.2 | 2782 | 38.9 | 3041 | 38.7 | 1581 | 38.5 |
| Less than 24 | 468 | 8.9 | 567 | 7.4 | 485 | 6.8 | 491 | 6.3 | 257 | 6.3 |
| 24 to 47 | 1466 | 28.0 | 1945 | 25.4 | 1,464 | 20.5 | 1450 | 18.5 | 626 | 15.2 |
| 48 or more | 1559 | 29.8 | 2452 | 32.0 | 2428 | 33.9 | 2875 | 36.6 | 1647 | 40.1 |
| **Diarrheal status** |  |  |  |  |  |  |  |  |  |  |
| No | 4713 | 89.9 | 7322 | 95.3 | 6756 | 94.3 | 7493 | 95.2 | 3903 | 94.8 |
| Yes | 528 | 10.1 | 359 | 4.7 | 411 | 5.7 | 379 | 4.8 | 212 | 5.2 |
| **Mother’s age** |  |  |  |  |  |  |  |  |  |  |
| 15 to 19 | 791 | 15.1 | 1029 | 13.4 | 1050 | 14.6 | 1020 | 13.0 | 405 | 9.8 |
| 20 to 34 | 3933 | 75.0 | 6005 | 78.2 | 5575 | 77.7 | 6257 | 79.4 | 3263 | 79.2 |
| 35 to 49 | 518 | 9.9 | 649 | 8.4 | 547 | 7.6 | 600 | 7.6 | 454 | 11.0 |
| **Maternal education** |  |  |  |  |  |  |  |  |  |  |
| No formal education | 1405 | 26.8 | 1539 | 20.0 | 1169 | 16.3 | 567 | 7.2 | 257 | 6.2 |
| Primary | 1658 | 31.6 | 2356 | 30.7 | 2008 | 28.0 | 2264 | 28.7 | 893 | 21.7 |
| Secondary | 1840 | 35.1 | 3252 | 42.3 | 3324 | 46.3 | 3843 | 48.8 | 2242 | 54.4 |
| Higher | 338 | 6.5 | 536 | 7.0 | 671 | 9.4 | 1202 | 15.3 | 729 | 17.7 |
| **Maternal employment status** |  |  |  |  |  |  |  |  |  |  |
| Unemployed | 3842 | 73.3 | 6983 | 90.9 | 5291 | 73.8 | 4683 | 59.5 | 3075 | 74.6 |
| Employed | 1399 | 26.7 | 700 | 9.1 | 1881 | 26.2 | 3194 | 40.6 | 1047 | 25.4 |
| **Maternal nutritional status** |  |  |  |  |  |  |  |  |  |  |
| Normal | 3160 | 60.4 | 4618 | 60.5 | 4265 | 59.7 | 4734 | 60.3 | 2330 | 56.6 |
| Malnourished | 2068 | 39.6 | 3021 | 39.5 | 2882 | 40.3 | 3116 | 39.7 | 1790 | 43.4 |
| **Father's education** |  |  |  |  |  |  |  |  |  |  |
| No formal education | 1838 | 35.1 | 2280 | 29.7 | 1850 | 25.8 | 1157 | 15.0 | 622 | 15.3 |
| Primary | 1467 | 28.0 | 2233 | 29.1 | 2160 | 30.1 | 2689 | 34.8 | 1240 | 30.5 |
| Secondary | 1349 | 25.8 | 2199 | 28.6 | 2191 | 30.6 | 2564 | 33.2 | 1438 | 35.4 |
| Higher | 581 | 11.1 | 965 | 12.6 | 970 | 13.5 | 1326 | 17.1 | 762 | 18.8 |
| **Total** | **5242** | **100.0** | **7683** | **100.0** | **7173** | **100.0** | **7877** | **100.0** | **4121** | **100.0** |

**Supplementary Figure 1** Trends in childhood CIAF in Bangladesh, 2007-2022

**Supplementary Table 3A** Disparities in childhood CIAF disaggregated by wealth quintile in Bangladesh (2007 to 2022)

| **Year** | **Concentration Index (CCI)** | **95% CI** | **P value** |
| --- | --- | --- | --- |
| 2007 | -0.220 | (-0.251, -0.190) | <0.001 |
| 2011 | -0.227 | (-0.252, -0.200) | <0.001 |
| 2014 | -0.241 | (-0.267, -0.220) | <0.001 |
| 2017-18 | -0.183 | (-0.208, -0.160) | <0.001 |
| 2022 | -0.175 | (-0.211, -0.140) | <0.001 |

**Supplementary Table 3B** Disparities in childhood CIAF disaggregated by place of residence in Bangladesh (2007 to 2022)

| **Year** | **Concentration Index (CCI)** | **95% CI** | **P value** |
| --- | --- | --- | --- |
| 2007 | -0.068 | (-0.09, -0.05) | <0.001 |
| 2011 | -0.055 | (-0.074, -0.04) | <0.001 |
| 2014 | -0.074 | (-0.094, -0.05) | <0.001 |
| 2017-18 | -0.052 | (-0.072, -0.03) | <0.001 |
| 2022 | -0.015 | (-0.043, 0.01) | 0.299 |

**Supplementary Table 3C** Disparities in childhood CIAF disaggregated by mother’s education in Bangladesh (2007 to 2022)

| **Year** | **Concentration Index (CCI)** | **95% CI** | **P value** |
| --- | --- | --- | --- |
| 2007 | -0.184 | (-0.213, -0.15) | <0.001 |
| 2011 | -0.204 | (-0.228, -0.18) | <0.001 |
| 2014 | -0.183 | (-0.208, -0.16) | <0.001 |
| 2017-18 | -0.193 | (-0.216, -0.17) | <0.001 |
| 2022 | -0.153 | (-0.187, -0.12) | <0.001 |

**Supplementary Table 3D** Disparities in childhood CIAF disaggregated by father’s education in Bangladesh (2007 to 2022)

| **Year** | **Concentration Index (CCI)** | **95% CI** | **P value** |
| --- | --- | --- | --- |
| 2007 | -0.193 | (-0.223, -0.16) | <0.001 |
| 2011 | -0.204 | (-0.228, -0.18) | <0.001 |
| 2014 | -0.196 | (-0.221, -0.17) | <0.001 |
| 2017-18 | -0.196 | (-0.221, -0.17) | <0.001 |
| 2022 | -0.129 | (-0.164, -0.09) | <0.001 |
